# Effect of frequency on crack growth in articular cartilage

**DOI:** 10.1016/j.jmbbm.2017.08.036

**Published:** 2018-01

**Authors:** H. Sadeghi, B.M. Lawless, D.M. Espino, D.E.T. Shepherd

**Affiliations:** Department of Mechanical Engineering, University of Birmingham, B15 2TT, UK

**Keywords:** Articular cartilage, Crack growth, Frequency, Osteoarthritis, Tensile

## Abstract

Cracks can occur in the articular cartilage surface due to the mechanical loading of the synovial joint, trauma or wear and tear. However, the propagation of such cracks under different frequencies of loading is unknown. The objective of this study was to determine the effect of frequency of loading on the growth of a pre-existing crack in cartilage specimens subjected to cyclic tensile strain. A 2.26 mm crack was introduced into cartilage specimens and crack growth was achieved by applying a sinusoidally varying tensile strain at frequencies of 1, 10 and 100 Hz (i.e. corresponding to normal, above normal and up to rapid heel-strike rise times, respectively). These frequencies were applied with a strain of between 10–20% and the crack length was measured at 0, 20, 50, 100, 500, 1000, 5000 and 10,000 cycles of strain. Crack growth increased with increasing number of cycles. The maximum crack growth was 0.6 ± 0.3 (mean ± standard deviation), 0.8 ± 0.2 and 1.1 ± 0.4 mm at frequencies of 1, 10 and 100 Hz, respectively following 10,000 cycles. Mean crack growth were 0.3 ± 0.2 and 0.4 ± 0.2 at frequencies of 1 and 10 Hz, respectively. However, this value increased up to 0.6 ± 0.4 mm at a frequency of 100 Hz. This study demonstrates that crack growth was greater at higher frequencies. The findings of this study may have implications in the early onset of osteoarthritis. This is because rapid heel-strike rise times have been implicated in the early onset of osteoarthritis.

## Graphical abstract

**Figure:**
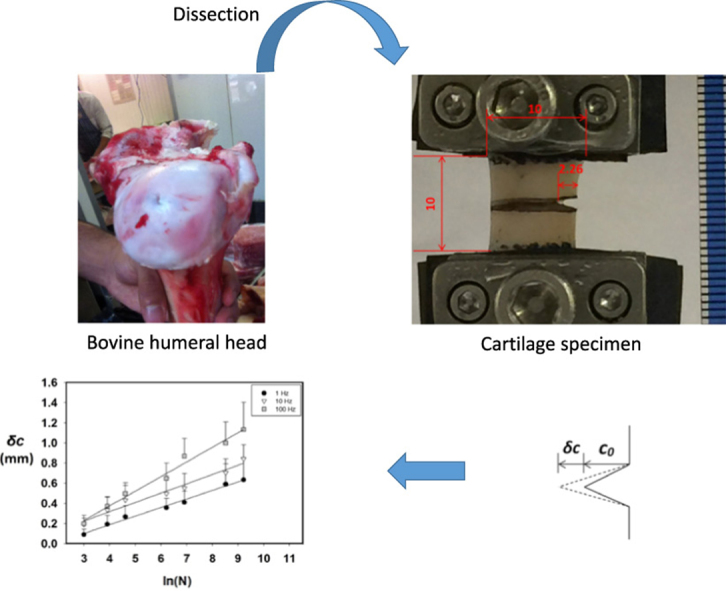

## 1 Introduction

Osteoarthritis (OA) is a degenerative, multifactorial disease. The most recognized symptom of this disease is pain that drives individuals to seek medical attention (Ayis and Dieppe, 2009). Approximately 27 million US adults and 8.5 million UK adults have clinical OA defined on the basis of symptoms and physical findings (National Collaborating Centre for Chronic Conditions, 2008, Lawrence et al., 2008). The significant disability associated with this disease is a great physical burden for affected individuals and an economic burden on the health-care system (Woolf and Pfleger, 2003). Although OA is considered to be a disease of the joint (Loeser et al., 2012), articular cartilage is central to the disease and its progression (Creamer and Hochberg, 1997). The disease involves a decrease in thickness and volume of the tissue (Cicuttini et al., 2004), in addition to an increase in the number and size of cartilage defects (Ding et al., 2005). This can be observed in both animal and human tissue (Clark, 1991). An important element in the disease is the fracture of cartilage, because once cartilage fractures, it has a limited ability to heal the cracks (Buckwalter et al., 1987). It has been hypothesised that these cracks grow with time as an important constituent of the development and progression of OA (Meachim, 1972).

Most testing of cartilage mechanical failure has been undertaken through quantifying the tensile strength of cartilage tissue (Akizuki et al., 1986) or one-dimensional tensile testing of cartilage samples (Roberts et al., 1986). However, cartilage fails by crack formation and fibrillation (Clark and Simonian, 1997). Qualitative measurements of the crack growth in slices of cartilage samples have introduced the concept of cartilage fracture as an important process in the degeneration of cartilage (Broom, 1986). Previous studies (Chin-Purcell and Lewis, 1996, Stok and Oloyede, 2003) have also suggested methods to measure the fracture toughness of cartilage. These studies came to the conclusion that cartilage failure *in vivo* involves the progressive growth of defects.

**Fig. 1:**
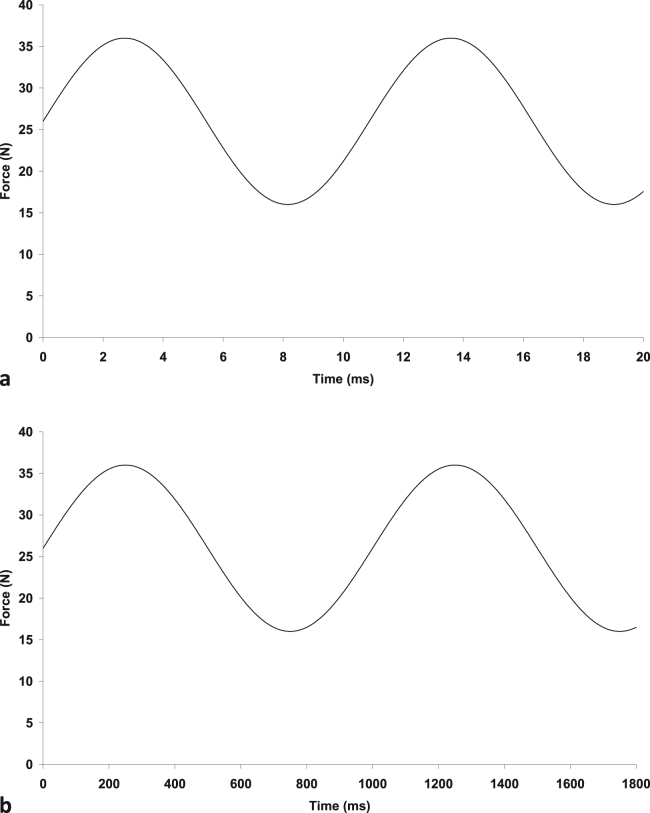
Sinusoidally varying force a) at 92 Hz (rise time 5.4 ms) b) at 1 Hz (rise time 500 ms).

Rapid heel-strike rise times during gait have been implicated in the early onset of OA in lower limb joints (Radin et al., 1986, Radin et al., 1991). Heel strike rise times in the normal population have been determined to be typically 100–150 ms (Shepherd and Seedhom, 1997). However, Radin et al. (1986) have shown that at heel-strike, some people exhibit a very high rate of loading with a distinct impulsive peak. These rapid heel-strikes take only 5–25 ms to reach a maximum force (Simon et al., 1981). The duration of the heel-strikes corresponds to loading frequencies of 3–5 Hz for normal and up to 90 Hz for impulsive heel-strike rise times (Fulcher et al., 2009). The effect of rapid heel-strike rise times on crack growth in articular cartilage can be investigated by subjecting cartilage specimens with an initial crack to frequencies representative to such rise times, as shown in Fig. 1. The rise time of the force is approximated by the time taken from the trough to the peak of the sine wave (Fulcher et al., 2009). That is

(1) $$t=\frac{1}{2f}$$

where *f* is the frequency of the sine wave. Thus, a sinusoidally varying force with a frequency of 92 Hz has been estimated as being representative of a rise time of 5.4 ms, and a frequency of 1 Hz as representative of a heel-strike with a rise time of 500 ms (Fulcher et al., 2009). It has been argued that rapid heel-strike rise times (e.g. 5 ms) lead to impulsive loading (Radin et al., 1991), which might be associated with a predisposition to OA.

Previous studies, on the fracture propagation, of articular cartilage have been focused on the effect of split line direction in tension (Sasazaki et al., 2006) or the effect of impact (Borrelli et al., 1997). However, the effect of frequency has been ignored. Previous studies (Fulcher et al., 2009, Sadeghi et al., 2015a, Espino et al., 2014) have hypothesised that the possibility of cartilage failure would increase with loading frequency because at higher frequencies the ability of the tissue to store energy increased. Therefore, at higher frequencies more energy is available to damage cartilage (Fulcher et al., 2009). Damage caused by increasing the loading frequency (or rate of loading) has been suggested to be different to the damage by increasing load only following comparisons between failure patterns from static loading tests (Fick and Espino, 2011, Fick and Espino, 2012). This has been demonstrated experimentally by increasing the loading frequency, from relevant gait (1 Hz) to an impulsive frequency (100 Hz), which resulted in more failure of the cartilage-on-bone specimen samples subjected to cyclic compression (Sadeghi et al., 2015b) and bending (Sadeghi et al., 2017). However, these studies focused on compression and bending, rather than tensile strains which have been implicated in the growth of superficial cartilage cracks during impact loading (Kelly and O’Connor, 1996). Therefore, it is currently unknown whether frequencies associated with rapid heel-strikes might also predispose articular cartilage to increased crack growth under purely tensile conditions.

This study aimed to investigate the effect of the variation of loading frequencies associated with relevant gait (1 Hz), above gait (10 Hz) and impulsive loading frequencies (100 Hz) on crack growth in bovine articular cartilage specimens subjected to tensile strains.

## 2 Methods

### 2.1 Specimen preparation

Ten bovine shoulder joints, aged between 18 and 24 months, were obtained from Dissect Supplies (King's Heath, Birmingham, UK). Bovine cartilage was used because it is an accepted model for human cartilage (Taylor et al., 2012) and the frequency-dependent viscoelastic trends of bovine articular cartilage have been shown to be consistent with those of human articular cartilage; this includes a similar frequency dependency and high-frequency plateau (Temple et al., 2016). Upon arrival in the laboratory, the humeral head, of each joint, was isolated. The humeral head was wrapped in tissue soaked in Ringer's solution (Sigma-Aldrich, Dorset, UK), sealed in a plastic bag and stored at – 40 °C. The influence of freeze-thaw treatment on the mechanical properties of articular cartilage was assumed to be negligible (Stok and Oloyede, 2003, Stok and Oloyede, 2007). On the day of testing, humeral heads were removed from storage, and allowed to thaw at room temperature. India ink (Loxley Art Materials, Sheffield, UK) was applied to the humeral heads to identify surface lesions (Meachim, 1972). The India ink was rinsed off and regions, without surface damage, were selected for testing. Three rectangular 40 × 20 mm specimens, which comprised of both bone and cartilage, were cut using a saw from the central load-bearing region of each humeral head.

The underlying bone was approximately 60 mm in thickness and was used to grip the specimens. A mandoline slicer (Mastrad inc., Paris, France) with a 1 mm gap was used to remove cartilage slices while it was still attached to the bone. Cartilage specimens had a maximum of 1 mm depth from the articulating surface towards the bone. In total 30 test specimens, consisting only of cartilage, were obtained from ten humeral heads.

**Fig. 2:**
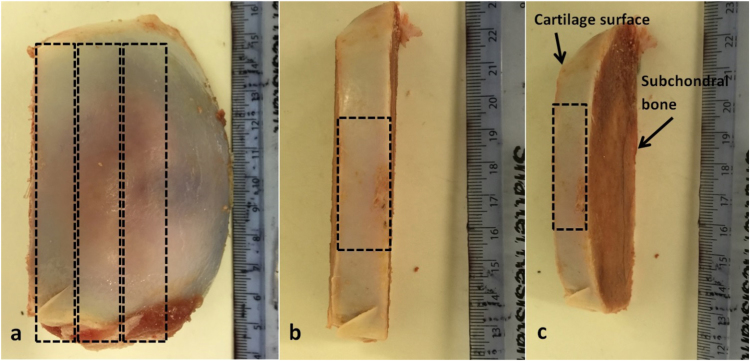
Specimen preparation. (a) Three sections of cartilage-on-bone were removed from each humeral head. (b) Top view of a cartialge-on-bone section. (c) Side view of a cartilage-on-bone section. Cuts were made in relation to the curvature of each humeral head. Cartilage sheets were obtained from the flattest surface of each cartilage-on-bone section using a mandoline slicer. The scale bar is in mm.

The final cartilage specimens for testing were then produced with dimensions of 20 × 10 mm using a 15 blade medical scalpel (Swann-Morton, Sheffield, UK). A digital Vernier calliper (Fisher Scientific, Leicestershire, UK) was used to measure and highlight an area of 10 × 10 mm and a 2.26 mm crack was cut into the middle of the specimen using a scalpel blade (Fig. 2). The length of the crack was based on the work of McCormack and Mansour (1998), where the initial crack length was 22.6% of the specimen width.

### 2.2 Mechanical testing

**Fig. 3:**
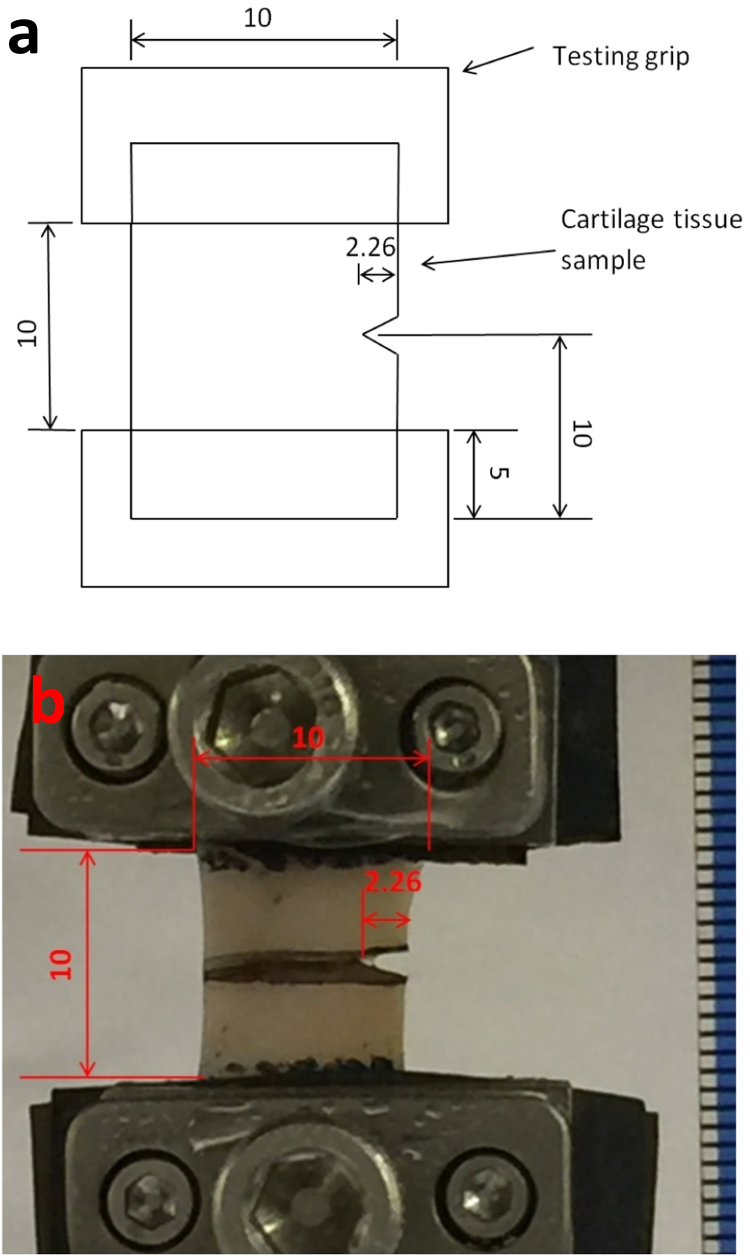
Mechanical testing. (a) Dimensions of the cartilage specimen. (b) Cartilage specimen placed in grips, ready for testing. All units are in mm.

Testing was performed using a Bose ElectroForce 3200 testing machine (Bose Corporation, Minnesota, USA; now, TA Instruments, New Castle, DE, USA) running WinTest 4.1 Software. Two custom-made grips were attached to the testing machine. Emery paper (120 grit) was fixed to the grips (McCormack and Mansour, 1998) and the cartilage specimen was secured by the tightening of screws (Fig. 3). A preload of 0.1 N was applied to the cartilage specimens to prevent the twisting of the specimens about the axial length (aligned with loading axis) while being tested. The actuator of the testing machine applied a sinusoidally varying tensile strain to the tissue specimen with a minimum of 10% and a maximum of 20% of the specimen length (10 mm) for 10,000 cycles. A block command function was used, to initially displace specimens to 15% of their gauge length; specimens were then held in this position for 5 s while an image was acquired. Images were taken using an Apple iPhone 6 Plus (Apple Inc, California, USA) operated under iOS 8 with Sony Exmor RS camera (8 megapixels, 1.5 focus pixels). A scale-bar was included in each image, positioned in the field of view. Images were acquired at 0, 20, 50, 100, 500, 1000, 5000 and 10,000 cycles. Specimens were tested at either 1 Hz, 10 Hz or 100 Hz; these test frequencies correspond to normal, above normal and up to rapid heel-strike rise times, respectively (Sadeghi et al., 2015b, Sadeghi et al., 2017). For the calculation of 95% confidence intervals, sample size for each frequency was 10 (*n* = 10) (Ranstam, 2012). To prevent dehydration, the tissue specimens were irrigated with Ringer's solution every 600 cycles.

### 2.3 Image and data analysis

Digital images were analysed using Image-J software (version 1.5, Rasband, W.S., U.S. National Institutes of Health, Bethesda, Maryland, USA). For each image, measurements were calibrated using the scale bar at the right-hand side of the frame. Lines were drawn manually along the crack length (*c*) for each image. The software was then used to measure the crack length (in mm) with a 0.1 mm precision. The crack growth (*δc*) was calculated from:

(2) $$\delta c=c-c_{0}$$

where *c* is the total crack length and *c*_*0*_ is the originally inserted crack length.

### 2.4 Statistical analysis

The crack length measurements were obtained from each image, therefore, 240 image analysis measurements were obtained in total. The progression of *δc* was analysed with respect to the number of cycles at the tested loading frequencies. All statistical analyses were undertaken using SigmaPlot (SYSTAT, San Jose, CA, USA). Regression analyses were performed to evaluate the significance of the curve fits. From the regression analyses, Mann-Whitney rank sum tests were performed to evaluate the difference of the coefficients and the constants between the three different frequencies. Statistical results were considered significant if *p* < 0.05.

## 3 Results

**Fig. 4:**
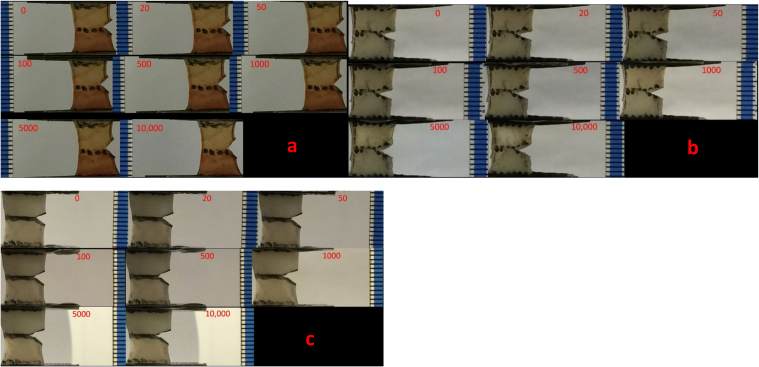
Specimen images taken at 0, 20, 50, 100, 500, 1000, 5000 and 10,000 cycle. Each specimen was subjected to a maximum strain of 20% undertaken at loading frequencies of a) 1 Hz, b) 10 Hz and c) 100 Hz. Increasing the loading frequency caused a higher crack growth in cartilage specimens. All units are in mm.

Images from three cartilage specimens, tested at 1, 10 and 100 Hz, with varying number of cycles are shown in Fig. 4. From Fig. 4, it can be observed that strain experienced by the specimens at higher frequency e.g. 100 Hz caused a greater crack growth.

**Fig. 5:**
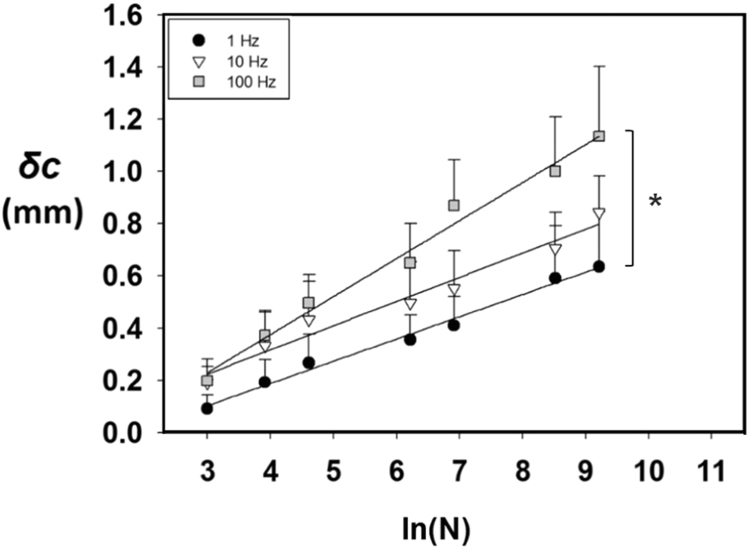
Mean crack growth (*δc*) against the natural logarithm of the number of cycles (*N*). Logarithmic curves (Eq. (3)) fitted the data points well. Error bars represent 95% confidence intervals for the samples. For clarity, only positive error bars are included. Asterisk denotes a significant difference at *p* < 0.05 between coefficients (*A*) of the curve fits.

Crack growth (*δc*) was found to increase significantly (*p* < 0.05) with an increasing number of loading cycles when the mean values of 10 specimens (per frequency) were plotted against the number of cycles (Fig. 5). Crack growth (*δc*) values at a frequency of 100 Hz were always larger compared to the crack growth at a frequency of 1 or 10 Hz. The trends for the crack growth (*δc*) against number of cycles (*N*) were described by the logarithmic curve fit

(3) δc=A(ln⁡(N))+B

where *A* and *B* are coefficients (Table 1). Coefficient (*A*) of the curve fits were found to increase from 0.08 to 0.14 mm at frequencies of 1–100 Hz, respectively. Only the coefficient (*A*) between 1 Hz and 100 Hz were significantly different; other comparisons were not significantly different (*p* > 0.05).

**Table 1:** Details of the constants from the mean crack growth against number of cycles curve fits. Units for *A* and *B* are in mm.

Frequency (Hz)	*A* (SE)	*B* (SE)	*R*^*2*^
1	0.08 (0.003)	− 0.15 (0.02)	0.98
10	0.09 (0.007)	− 0.05 (0.04)	0.94
100	0.14 (0.008)	− 0.2 (0.05)	0.90

The correlation between crack growth and number of cycles is described by logarithmic curve fits for each loading frequency. Mean crack growth against number of cycles curve fits were statistically significant (*p* < 0.001) for all frequencies. *SE* is the standard error of the coefficients *A* and *B. R*^*2*^ is a squared correlation coefficient and shows how well the line fits the data points.

**Fig. 6:**
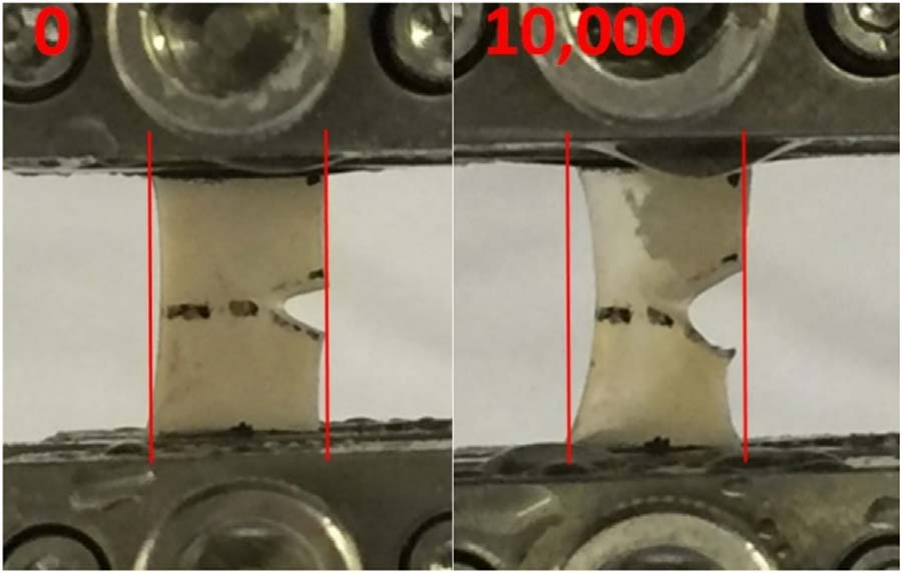
Sample images taken from cartilage specimen number 8, tested at frequency of 1 Hz at the initial testing position and following 10,000 cycles. Vertical lines were inserted in each image to show the displacement of the sides of the cartilage specimen relative to their initial position.

All ten specimens were found to undergo extensive necking during testing at the frequency of 1 Hz (Fig. 4a, Fig. 6). However, necking occurred at a lower degree to the specimens tested at 10 or 100 Hz (Fig. 4b, c). Necking was also found to progress continually with increased number of cycles from qualitative observations (Fig. 4a, Fig. 6).

## 4 Discussion

The results from this study demonstrate how pre-existing cracks in articular cartilage samples grow when subjected to cyclic strains at different frequencies. In the current study, crack growth was found to increase with an increasing number of cycles. Crack growth rate was also found to be greater at higher frequencies. This is a new observation because controlled crack growth experiments have not been used to explain the influence of frequency on the growth of pre-existing cracks in cartilage off-bone samples under tension. Initiation and progression of damage can cause failure in the articular cartilage of joints. Understanding the mechanism associated with the failure of articular cartilage is important to help identify strategic solutions which curtail such failure mechanisms. Crack growth in cartilage specimens, where cracks are initiated through the depth of cartilage specimens and allowed to propagate at different frequencies could help to further understand the link between mechanical loading and cartilage failure. Experimental data previously obtained from cartilage samples have shown that pre-existing cracks can grow under tension (Stok and Oloyede, 2003, Stok and Oloyede, 2007). In our current study, the crack length increased with increases in the frequency between 1, 10 and 100 Hz, independent of load.

The findings of the current study are consistent with those of Sadeghi et al. (2015b), where subjecting cartilage-on-bone samples to 10,000 cycles of compressive loads using a metal indenter (5.2 mm diameter), resulted in crack formation on the articular surface of tissue samples. A follow-up study (Sadeghi et al., 2017) subjected beam-shaped cartilage-on-bone samples to cyclic three-point bending. It also showed that increasing the loading frequency from normal and above gait (1 and 10 Hz) to impulsive loading frequencies (50 and 100 Hz) reduced the ability of the specimen samples to resist fracture. However, those studies focused on cartilage-on-bone, under compression and three-point bending, not purely tensile loading.

In the present study, higher crack growth rate of specimen samples occurred at higher frequencies. This could correspond to cartilage approaching a glass transition when subjected to dynamic loading at frequencies above 10 Hz. Cartilage changes from soft tissue to “glass-like” material (Fulcher et al., 2009) that could result in the fast growth of cracks in cartilage samples when subjected to impulsive frequencies (Kelly and O’Connor, 1996). Other studies (Fulcher et al., 2009, Sadeghi et al., 2015a, Espino et al., 2014, Pearson and Espino, 2013) have also suggested that there might be an increase in cartilage damage with increasing loading frequency. They noted that it might be based on the frequency dependency of viscoelastic properties of cartilage. The suggestion was consistent with the reported increase in energy under impact loading *in vitro* was also suggested to cause cartilage fracture (Jeffrey and Aspden, 2006) and measurements of hysteresis (energy dissipation) following drop-tower tests showed that hysteresis could increase with increased loading velocity (Edelsten et al., 2010). Fulcher et al. (2009) hypothesised that cartilage underwent a glass transition due to the alteration in frequency-dependent trends of storage and loss moduli. At higher frequencies, the ability of the tissue to store energy for elastic recoil increased. However, the ability of the tissue to dissipate energy remained the same. It was, thus, suggested that if the energy available for storage exceeded a certain level it might induce damage to the cartilage (Fulcher et al., 2009). The dependence of storage modulus on frequency, in which the storage modulus increases but then levels out to a plateau, is characteristic of a material undergoing a glass transition (Ferry, 1980).

In our current study, it was found that specimens subjected to tensile strain at a frequency of 1 Hz underwent necking, while this effect happened to a lesser degree for specimens tested at frequencies of 10 or 100 Hz. Necking represents a lateral displacement, of the intact side of cartilage, relative to its initial length along the axis of loading. This observation has previously been reported in full thickness cartilage samples following tensile loading (Stok and Oloyede, 2003, Stok and Oloyede, 2007). These studies suggested that as cartilage is loaded in tension, the collagen fibres within the solid matrix align and stretch along the axis of loading. Such necking might be due to the pull of the collagen fibres in response to the tensile strain. However, this effect is more evident in samples tested at 1 Hz as opposed to 100 Hz. Collagen is arranged to provide reinforcing to a highly hydrated proteoglycan gel in cartilage tissue (Aspden and Hukins, 1981). Viscoelastic properties of articular cartilage are associated with the stress transfer mechanism during gel-collagen interaction (Goh et al., 2010). Thus, the difference in the behaviour of cartilage samples at the tested frequencies could be the consequence of the variation of the interactions between the structural components of the tissue. This could also be related to before/after glass transition (Fulcher et al., 2009).

Flachsmann et al. (2006) subjected cartilage-on-bone samples to compression using an indenter (8 mm diameter). They found that introducing cracks about 1 mm in length in the superficial layer of cartilage reduced the compressive strength to less than half of the value measured from samples without cracks. Increased compression at the edge of a surface defect (Braman et al., 2005) as well as increased cartilage failure in a joint with a pre-existing defect have also been described in the literature (Hollander et al., 1995). These findings are in agreement with the current study that showed a pre-existing crack could grow in a cartilage specimen subjected to cyclic strains, which may lead to the complete failure of the specimen. Quinn et al. (2001) subjected cartilage-on-bone samples to impact loading at varying strain rates between 3 × 10^−5^ and 0.7 s^−1^, to maximum stresses in the range 3.5–14 MPa, respectively. At the higher strain rates tissue cracks were observed particularly near the superficial layer. However, at lower strain rates no signs of damage were observed. This is consistent with the findings of this study that showed crack growth rate is higher at higher loading frequencies. However, these studies were of cartilage-on-bone samples and tested in compression.

Most studies on the tensile failure of cartilage (McCormack and Mansour, 1998, Weightman, 1976, Weightman et al., 1978, Kempson, 1982, Kempson, 1991) have been concerned with variations in properties among joints, the effects of repeated load, and age. The tensile loading of human cartilage samples *in vitro* was shown to cause surface fibrillation (Weightman, 1976). Cartilage samples from the femoral head were subjected to cyclic tensile stress by Weightman et al. (1978). They found that the resistance of the specimen samples decreased with age. Other studies (Kempson, 1982, Kempson, 1991) documented changes including a decrease in tensile strength and stiffness of cartilage tissue which might make the tissue more predisposed to injury and development of degeneration. While McCormack and Mansour (1998) found that a sufficient number of compressive load cycles under an average stress of 3.2 MPa, applied to the cartilage surface *in situ,* caused a decrease in tensile strength. After 64,800 cycles of compressive loading their results showed no change in the tensile strength of cartilage, but after 97,200 cycles, tensile strength was reduced significantly. The difference is that McCormack and Mansour (1998) tested cartilage in tension following compressive cycles of loading. However, it demonstrated that following cyclic loading, cartilage was prone to failure under tension which is in agreement with the findings of this study. The current study, however, goes a step further by demonstrating that the frequency of loading is variable in the tensile failure of cartilage.

In a comparable study, subjecting bovine cartilage to 10,000 cycles of maximum stress of 4.2 MPa at frequency of 10 Hz and a lower stress of 2.8 MPa but at a higher frequency of 100 Hz, produced cracks on the cartilage surface (Sadeghi et al., 2015b). In the current study the number of cycles was kept constant to analyse the effect of altering the loading frequency on crack growth while specimens were subjected to an average 15% strain. Maximum physiological cartilage strains have been measured to be approximately 12% which are consistent with the dynamic strain used in the current study (Chan et al., 2016). However, cartilage *ex vivo* studies have often exceeded these strains assessing cartilage at strains of up to 40% (Demarteau et al., 2006) and 60% (Edelsten et al., 2010).

Our current study demonstrates that the frequency at which a joint is loaded is an additional factor when assessing crack growth and cartilage failure. Previous studies have shown that high tensile stresses are generated in the knee in flexion (Minns et al., 1979) and these stresses arise in the surrounding regions of the loaded surfaces in contact in the joint (Kelly and O’Connor, 1996). Tensile failure of cartilage has been of interest, because it was suggested that vertical cracks in cartilage were initiated by tensile stresses on the articular surface (Eberhardt et al., 1991). Fracture mechanics of cartilage have been studied extensively (Chin-Purcell and Lewis, 1996, Stok and Oloyede, 2003, Stok and Oloyede, 2007) by measuring fracture toughness, or the ability of the cartilage to resist crack growth. Stok and Oloyede, 2003, Stok and Oloyede, 2007) and Chin-Purcell and Lewis (1996) studied crack growth in cartilage with tensile loading, while Wang et al. (2011) investigated the strain distribution at compressive loading. Chin-Purcell and Lewis (1996) measured the fracture toughness of cartilage using energy based methods. This study showed that cartilage quickly distributes loads via crack growth when subjected to instantaneous loads to avoid stress concentration.

### 4.1 Limitations and future work

The initial crack was introduced in the cartilage specimens in this study and the growth of such cracks was observed across the area of the cartilage specimens with respect to increasing the number of cycles of loading. This is distinct from the methods used by comparable studies (Stok and Oloyede, 2003, Stok and Oloyede, 2007) in which the crack growth was investigated through the depth of cartilage specimens. Propagation across cartilage is relevant as shown by Fick (2013) who found that loading was distributed to adjacent areas under compression. This indicates that crack propagation could occur across the area of cartilage not only through the depth; which has been confirmed through this present study. Observations on necking have previously focused on necking through the depth of cartilage specimens (Stok and Oloyede, 2003, Stok and Oloyede, 2007). However, in the current study, observations were made on necking perpendicular to the cartilage depth. Although this could make the results of this study less physiologically relevant, it also demonstrates the role of collagen recruitment during loading and propagation of failure through the depth and perpendicular to the 20 mm edge of cartilage specimens. It was not the aim of the current study to distinguish between different fracture mechanisms of crack growth in cartilage. However, the specimens in this study underwent repeated cyclical loading to understand the crack propagation in cartilage.

Specimens used in this study had a maximum of 1 mm depth from the articulating surface towards the bone. These specimens likely include superficial and transition-zones as well as the middle and deep zones, in part or in full, given that bovine humeral head varies from 0.8 to 1.6 mm (Töyräs et al., 2001). One of the other limitations of this study was that cartilage has zonal variations of collagen orientation across its area (Fick, 2013) which was not accounted for in our study. There also may have been limited repeatability of between specimens due to testing along or perpendicular to split-line directions, and due to some cartilage specimens being full depth and others not; as collagen fibril orientation varies through its depth (Aspden and Hukins, 1981, Minns and Steven, 1977). These could limit the wider applicability of our findings. Regardless, this study found a clear trend of increased crack propagation across the surface with increased frequency. This finding opens up the potential for future studies which asses how frequency of loading and failure interact with collagen orientation.

## 5 Conclusion

Crack growth increased with the number of cycles of loading. The crack growth rate of cartilage samples was greater at higher frequencies. The variation of crack growth of cartilage specimen samples at loading frequencies associated with normal (1 Hz), above normal (10 Hz) and up to rapid heel-strike rise times (100 Hz) may have implications in the early stages of OA.

## Competing interests

The authors declare that they have no competing interests.
